# Drug-eluting stents or coronary artery bypass grafting for unprotected left main coronary artery disease: a meta-analysis of four randomized trials and seventeen observational studies

**DOI:** 10.1186/1745-6215-14-133

**Published:** 2013-05-08

**Authors:** Qing Li, Zhi Zhang, Rui-Xing Yin

**Affiliations:** 1 Department of Cardiology, Institute of Cardiovascular Diseases, the First Affiliated Hospital, Guangxi Medical University, 22 Shuangyong Road, Nanning 530021 Guangxi, People’s Republic of China; 2 Department of Cardiology, Shanghai First People’s Hospital, Shanghai Jiao Tong University School of Medicine, Shanghai, 200080, People’s Republic of China

**Keywords:** Drug-eluting stents, Coronary artery bypass grafting, Unprotected left main coronary artery disease, Safety and efficacy, Meta-analysis

## Abstract

**Background:**

The clinical application of drug-eluting stents (DES) or coronary artery bypass grafting (CABG) for unprotected left main coronary artery disease (ULMCAD) is still controversial. The purpose of this meta-analysis was to compare the safety and efficacy between DES and CABG for ULMCAD.

**Methods:**

Databases of MEDLINE, EMBASE and the Cochrane Library were systematically searched.

**Results:**

Twenty-one studies with 8,413 patients were included in this meta-analysis. The risk was lower in DES than in CABG groups at the early outcomes of death (risk ratio (RR): 0.49, 95% confidence interval (CI): 0.30–0.78), cerebrovascular events (RR: 0.19, 95% CI: 0.08–0.45) and composite endpoint (RR: 0.53, 95% CI: 0.40–0.70); death after 2 years (RR: 0.81, 95% CI: 0.66–0.99), 4 years (RR: 0.69, 95% CI: 0.53–0.90), 5 years (OR: 0.76, 95% CI: 0.61–0.95) and their total effect (RR: 0.79, 95% CI: 0.71–0.87); composite endpoint 1 year (RR: 0.69, 95% CI: 0.58–0.83), 4 years (RR: 0.69, 95% CI: 0.53–0.88), 5 years (RR: 0.74, 95% CI: 0.59–0.92) and their total effect (RR: 0.78, 95% CI: 0.71–0.85). There were no significant differences in the risk for the early outcomes of myocardial infarction (RR: 0.97, 95% CI: 0.68–1.38), death 1 year (OR: 0.81, 95% CI: 0.57–1.15) and 3 years (OR: 0.85, 95% CI: 0.69–1.04), composite endpoint of 2 years (RR: 0.88, 95% CI: 0.72–1.09) and 3 years (RR: 0.87, 95% CI: 0.73–1.04). Nonetheless, there was a lower risk for revascularization associated with CABG from 1 to 5 years and their total effect (RR: 3.77, 95% CI: 3.35–4.26). There was no difference in death, myocardial infarction, cerebrovascular events or revascularization at 1 year between RCT and observational groups.

**Conclusions:**

Our meta-analysis indicates that DES has higher safety but higher revascularization than CABG in patients with ULMCAD in the 5 years after intervention.

## Background

As is well known, approximately 4 to 9% of patients undergoing diagnostic coronary angiography [1] are found to have unprotected left main stenosis which has been shown to portend high mortality [2,3]. Percutaneous coronary intervention (PCI) involving drug-eluting stents (DES) have increasingly been used to treat unprotected left main coronary artery disease (ULMCAD) in recent years, although coronary artery bypass grafting (CABG) has been the treatment of choice historically [4,5]. One of the main limitations of PCI for ULMCAD is in-stent restenosis and the need for repeat revascularization, especially in bare-metal stents [6,7]; therefore, the European Society of Cardiology guidelines and American Heart Association guidelines suggest that PCI for ULMCAD should be only reserved for those who are poor candidates for CABG [8]. However, several meta-analyses [9-12] of DES *versus* CABG for ULMCAD showed that the results are controversial, and many new clinical trials have been published in recent years [13-16]. Therefore, it is necessary to conduct a new meta-analysis and to assess the safety and efficacy of DES and CABG among patients with ULMCAD in the early outcomes (≤30 days or in-hospital) and 1 to 5 years follow-up, and it is also necessary to compare the difference in safety and efficacy of DES and CABG between RCT and observational groups.

## Methods

### Search strategy

The data of this meta-analysis were obtained from the following sources: MEDLINE via PubMed (from 1950 to June 2012), EMBASE (June 1980 to June 2012) and the Cochrane Library database (Cochrane Central Register of Controlled Trials, from 1991 to June 2012). The following keywords were used: “coronary artery bypass”, “drug-eluting stent”, “paclitaxel-eluting stent”, “sirolimus-eluting stent”, and “left main coronary artery”. The above search strategy described was used to obtain titles and abstracts of studies that may have been relevant to this review. The titles and abstracts were screened independently by two authors (Q Li and Z Zhang), who discarded studies that were not applicable. When multiple reports from the same patients were found, only the study with the most complete data set was included in the meta-analysis. However, duplicate patients of different articles that have different types of data of outcomes were included both. Any disagreements were arbitrated by discussion with a third reviewer (RX Yin).

### Included and excluded studies

Studies were included in this meta-analysis if they met the following criteria: 1) clinical trials published in peer-reviewed journals with full available text in English; 2) clinical trials comparing CABG with DES for LMCAD; 3) reporting at least one relevant clinical endpoint including revascularization, myocardial infarction, cerebrovascular events, death or the composite endpoint (death, myocardial infarction, or cerebrovascular events); and 4) follow-up duration ≥30 days. Excluded studies: 1) studies using only bare-metal stents or mixtures of bare-metal stents and DES but not comparing DES with CABG separately in the PCI group were excluded from this study; 2) studies in which it was not possible to extract data from the published results as well as those studies that did not report appropriate outcomes were also excluded.

### Types of outcome measures

The safety endpoints of this meta-analysis were death, cerebrovascular events, myocardial infarction and the composite endpoint of death, myocardial infarction or cerebrovascular events. The efficacy endpoint was revascularization. Death was defined as death from any cause. Myocardial infarction included Q-wave and non-Q-wave myocardial infarction. Cerebrovascular events included ischemic attacks, stroke and reversible ischemic neurological deficits. Revascularization was the need for repeated CABG or PCI.

### Data extraction and management

Two investigators independently extracted data according to the author details and the following information was extracted from each study: methodological quality, first author, the year of publication, number of patients in each group (CABG or DES), baseline characteristics, interventions, outcomes, and duration of follow-up. Otherwise, probabilities of death or other endpoints were estimated from published Kaplan-Meier survival curves. Discrepancies were resolved by discussion. When repeated publications of the same trial were identified, data were extracted from the repeated publications and reported as a single trial.

### Quality of the evidence recommendations methodology

The evidence recommendations in our meta-analysis were graded according to the Grading of Recommendations Assessment Development and Evaluation (GRADE) system by Grade software [17]. The quality of the evidence was classified in four levels: high (⊕⊕⊕⊕), moderate (⊕⊕⊕⊝), low (⊕⊕⊝⊝) or very low (⊕⊝⊝⊝).

### Statistical analysis

We carried out statistical analysis by the Review Manager software 5.1.0 (updated in March 2011 by the Cochrane Collaboration). Dichotomous outcomes of individual studies were expressed as risk ratio (RR) with 95% confidence intervals (CI). The pooled effects were calculated using fixed-effects models when there was no significant heterogeneity but the random effects model was analyzed to ensure robustness of the model chosen and susceptibility to outliers, or using random effects models when there was significant heterogeneity. The fixed effects model was analyzed to ensure robustness of the model chosen and susceptibility to outliers. The point estimate of the RR was considered statistically significant at the 2-tailed *P* ≤0.05 level. Heterogeneity was analyzed using a χ^2^ test on N-1 degrees of freedom [18]. *I*^2^ values of 25%, 50% and 75% correspond to low, medium and high levels of heterogeneity, respectively. Subgroup analysis was used to explore possible sources of heterogeneity (*e.g.*, duration of follow-up, type of outcomes and study quality). Sensitivity analyses were performed omitting a single study at a time or analyzing another model chosen. If enough studies were identified, funnel plots were used to investigate reporting biases [19]. The baseline characteristics were analyzed with χ^2^ test for categorical variables.

## Results

### Characteristics of included studies

**Figure 1:**
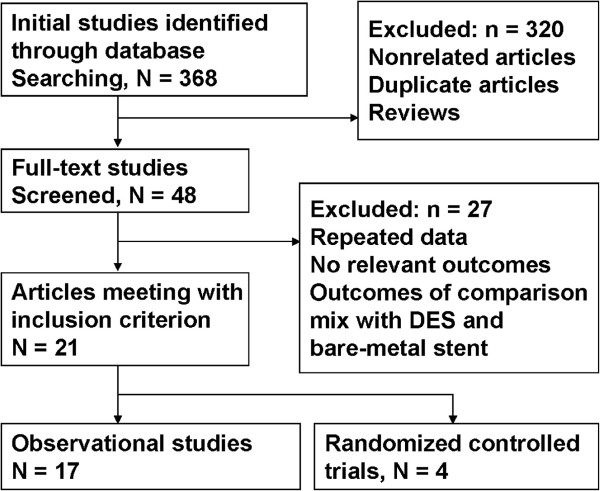
Flow chart showing study selection process.

Twenty-one studies met our criteria for inclusion in the meta-analysis (Figure 1). Four studies were randomized controlled trials [13,15,20,21] and seventeen studies were observational studies [4-8,14,16,22-36]. Several studies may have had duplicate patients but they had different types data of outcomes, *e.g.*, one study [20] included death outcomes but another [13] did not. A total of 8,413 patients were included in the analysis. There were 4,731 patients who received CABG and 3,682 patients who received PCI with DES. The main characteristics of the studies are shown in Table 1.

**Table 1:** Main characteristics of included studies

**Study**	**Year**	**Patients (DES/CABG)**	**Study year**	**Study design**	**Age (years) (DES/CABG)**	**Outcome**	**Follow-up period**
**Lee et al.**[27]	**2006**	**50/123**	**2003–2006**	**Observational**	**70/72**	**death, MI, TVR, stroke**	**1**
**Chieffo et al.**[24]	**2006**	**107/142**	**2002–2004**	**Observational**	**68/64**	**death, MI, TVR, stroke, MACCE**	**1**
**Palmerini et al.**[29]	**2007**	**98/161**	**2003–2006**	**Observational**	**78/81**	**death, MI, TVR**	**2**
**Sanmartin et al.**[32]	**2007**	**96/245**	**2000–2005**	**Observational**	**66/66**	**death, MI, TVR, stroke, MACCE**	**1**
**Makikallio et al.**[28]	**2008**	**49/238**	**2005–2007**	**Observational**	**72/70**	**death, MI, TVR, stroke, MACCE**	**1**
**White et al.**[35]	**2008**	**67/67**	**2003–2007**	**Observational**	**72/68**	**death, MACCE**	**2**
**Seung et al.**[33]	**2008**	**396/396**	**2003–2006**	**Observational**	**66/66**	**death, TVR, MACCE**	**3**
**Boudriot et al.**[20]	**2008**	**79/80**	**2003–2007**	**RCT**	**69/66**	**death, MI, TVR, MACCE**	**1**
**Cheng et al.**[22]	**2009**	**94/216**	**2000–2007**	**Observational**	**67/68**	**death, TVR, MACCE**	**3**
**Ghenim et al.**[25]	**2009**	**105/106**	**2004–2007**	**Observational**	**80/79**	**TVR, MACCE**	**1**
**Morice et al.**[21]	**2010**	**357/348**	**2005–2007**	**RCT**	**66/65**	**death, MI, TVR, stroke**	**1**
**Chieffo et al.**[23]	**2010**	**107/142**	**2002–2004**	**Observational**	**63/67**	**death, MI, TVR, stroke, MACCE**	**5**
**Kang et al.**[26]	**2010**	**205/257**	**2003–2006**	**Observational**	**64/65**	**death, MI, TVR, stroke, MACCE**	**3**
**Park et al.**[31]	**2010**	**784/690**	**2003–2006**	**Observational**	**63/64**	**death, TVR, MACCE**	**5**
**Park et al.**[30]	**2010**	**176/219**	**2003–2004**	**Observational**	**61/62**	**death, TVR, MI, stroke**	**5**
**Shimizu et al.**[34]	**2010**	**64/89**	**2004–2007**	**Observational**	**71/70**	**MI, TVR, stroke**	**1**
**Wu et al.**[36]	**2010**	**131/245**	**2003–2006**	**Observational**	**62/64**	**death, TVR, MACCE**	**4**
**Boudriot et al.**[13]	**2011**	**100/101**	**2003–2009**	**RCT**	**66/69**	**death, MI, TVR, MACCE**	**1**
**Park et al.**[15]	**2011**	**300/300**	**2004–2009**	**RCT**	**61/62**	**death, MI, TVR, stroke**	**2**
**Caggegi et al.**[14]	**2011**	**222/361**	**2002–2010**	**Observational**	**67/66**	**death, MI, TVR**	**1**
**Rittger et al.**[16]	**2011**	**95/205**	**2004–2007**	**Observational**	**71/68**	**death, stroke, TVR**	**2**

MACCE: Major adverse cardiac cerebrovascular events; MI: Myocardial infarction; TVR: Target vessel revascularization.

### Baseline characteristics of the trials

The baseline clinical characteristics between the PCI and CABG groups are detailed in Table 2. There were no significant differences in the prevalence of hypertension, current smoking, diabetes mellitus, previous stroke, and chronic renal failure between the two groups (*P* >0.05 for all). The proportions of females and previous PCI were lower but the prevalence of hyperlipidemia, previous myocardial infarction and right coronary artery disease were higher in CABG than in PCI groups (*P* <0.05 for all).

**Table 2:** Baseline clinical characteristics

**Characteristic**	**PCI**	**CABG**	***P *** **(*χ***^**2**^**)**
Number	3682	4731	
Female/Sample size	756/2644	862/3725	<0.001
Hypertension/Sample size	1759/2858	2529/4008	0.190
Current smoking/Sample size	893/2763	1306/3804	0.088
Hyperlipidemia/Sample size	1341/2809	1903/3771	0.029
Diabetes mellitus/Sample size	959/2858	1374/4009	0.536
Previous myocardial infarction/Sample size	320/2537	522/3401	0.003
Previous stroke/Sample size	204/1882	254/2376	0.876
Previous PCI/Sample size	415/2022	379/2809	<0.001
CRF/Sample size	156/2614	237/3465	0.171
RCA/Sample size	1009/1941	1699/2509	<0.001

Comparison of preoperative variable in DES and CABG patients. All variables come from the individual studies included. RCA: Right coronary artery; CRF: Chronic renal failure.

### Clinical outcomes

#### The early outcomes (≤30 days or in-hospital)

**Figure 2:**
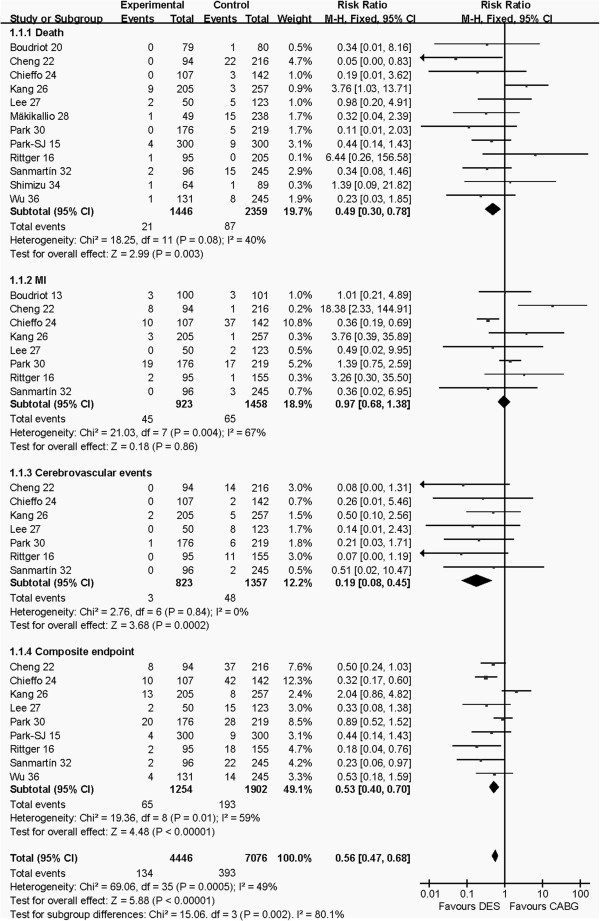
Comparison of the early outcomes (≤30 days or in-hospital) between PCI and CABG groups.

The early outcomes of DES and CABG groups and the pooled effects are shown in Figure 2. Pooled effects indicated that CABG group had higher risk of death (RR: 0.49, 95% CI: 0.30–0.78, *P* = 0.003), cerebrovascular events (RR: 0.19, 95% CI: 0.08–0.45, *P* = 0.0002) and composite endpoint (RR: 0.53, 95% CI: 0.40–0.70, *P* <0.00001) than the PCI group. There was no difference in myocardial infarction (RR: 0.97, 95% CI: 0.68–1.38, *P* = 0.86) between CABG and PCI groups.

#### Death after 1 to 5 years post-operation

**Figure 3:**
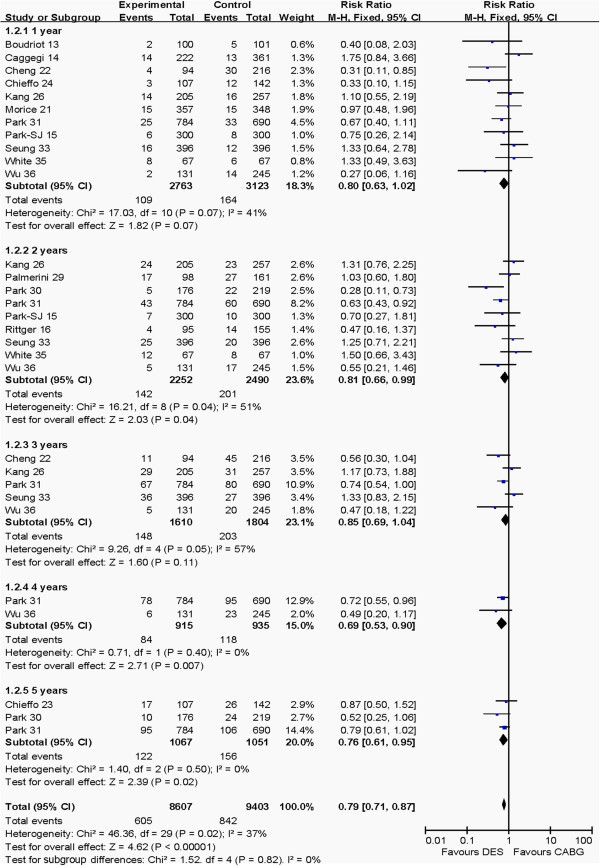
Comparison of the outcome of death from 1 to 5 years post-operation between PCI and CABG groups.

Death after 1 to 5 years post-operation between the CABG and PCI groups is shown in Figure 3. Pooled effects showed that CABG group had higher risk of death than the PCI group after 2 years (RR: 0.81, 95% CI: 0.66–0.99, *P* = 0.04), 4 years (RR: 0.69, 95% CI: 0.53–0.90, *P* = 0.007), 5 years (OR: 0.76, 95% CI: 0.61–0.95, *P* = 0.02) and total pooled outcome (RR: 0.79, 95% CI: 0.71–0.87, *P* <0.00001). There was no difference in deaths at 1 year (RR: 0.80, 95% CI: 0.63–1.02, *P* = 0.07) and 3 years (OR: 0.85, 95% CI: 0.69–1.04, *P* = 0.11) between the CABG and PCI groups.

#### Composite endpoint at 1 to 5 years post-operation

**Figure 4:**
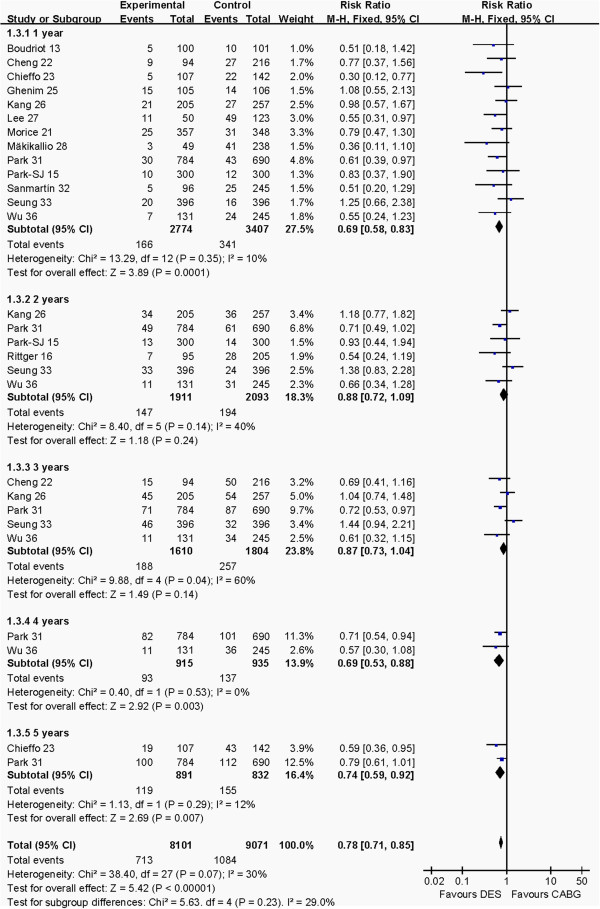
Comparison of the outcome of composite endpoint of death, myocardial infarction and cerebrovascular events from 1 to 5 years post-operation between PCI and CABG groups.

The outcomes of composite endpoint of death, myocardial infarction and cerebrovascular events at 1 to 5 year post-operation between CABG and PCI groups are detailed in Figure 4. Pooled effects showed that CABG group had higher composite endpoint risk than PCI group after 1 year (RR: 0.69, 95% CI: 0.58–0.83, *P* = 0.0001), 4 years (RR: 0.69, 95% CI: 0.53–0.88, *P* = 0.003), 5 years (RR: 0.74, 95% CI: 0.59–0.92, *P* = 0.007) and total pooled outcome (RR: 0.78, 95% CI: 0.71–0.85, *P* <0.00001). There was no difference in composite endpoint at 2 years (RR: 0.88, 95% CI: 0.72–1.09, *P* = 0.24) and 3 years (RR: 0.87, 95% CI: 0.73–1.04, *P* = 0.14) between the CABG and PCI groups.

#### Revascularization at 1 to 5 years post-operation

**Figure 5:**
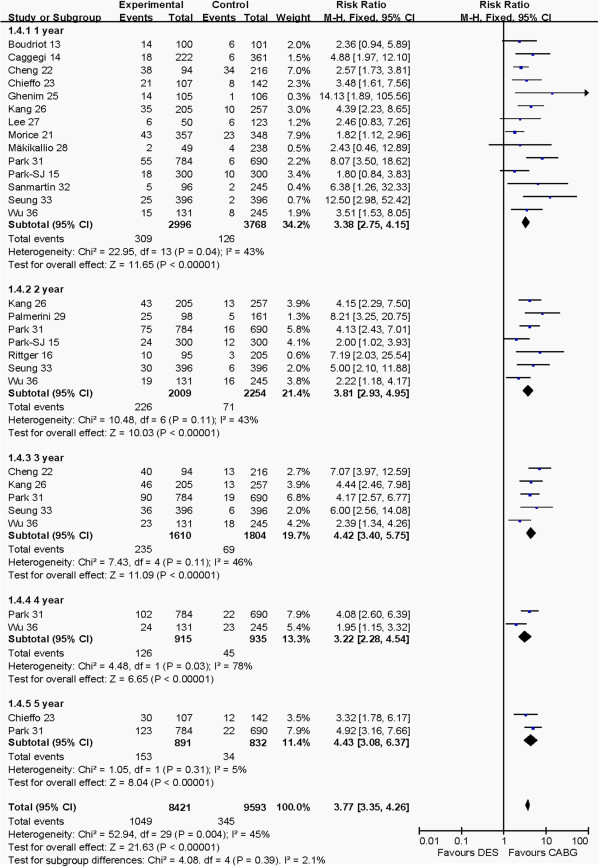
Comparison of the revascularization at 1 to 5 years post-operation between PCI and CABG groups.

The outcomes of revascularization at 1 to 5 years post-operation between PCI and CABG groups are shown in Figure 5. Pooled effects showed that PCI group had higher revascularization risk than CABG group at 1 year (RR:3.38, 95% CI: 2.75–4.15, *P* <0.00001), 2 years (RR: 3.81, 95% CI: 2.93–4.95, *P* <0.00001), 3 years (RR: 4.42, 95% CI: 3.40–5.75, *P* <0.00001), 4 years (RR: 3.22, 95% CI: 2.28–4.54, *P* <0.00001) and 5 years (RR: 4.43, 95% CI: 3.08–6.37, *P* <0.00001), and total pooled outcome (RR: 3.77, 95% CI: 3.35–4.26, *P* <0.00001).

### Outcomes at 1 year between RCT and observational groups

**Figure 6:**
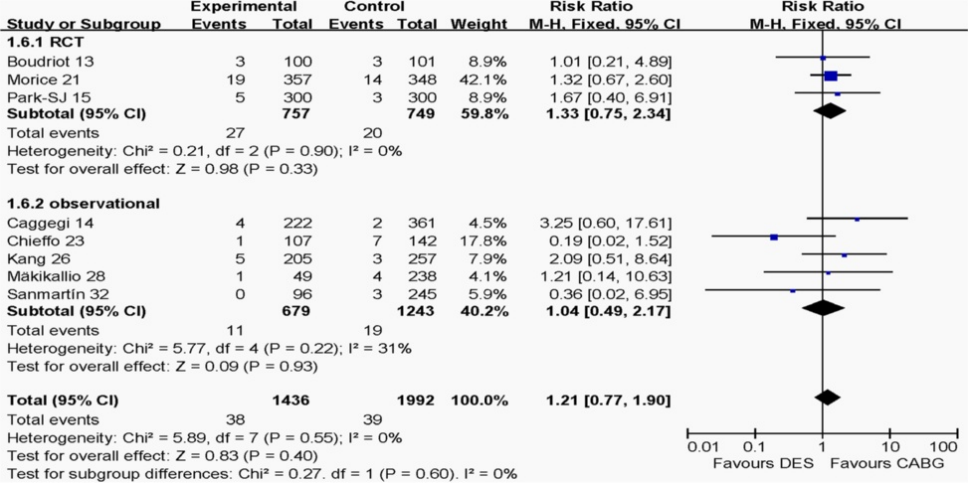
Comparison of DES and CABG of myocardial infarction at 1 year post-operation between RCT and observational groups.

**Figure 7:**
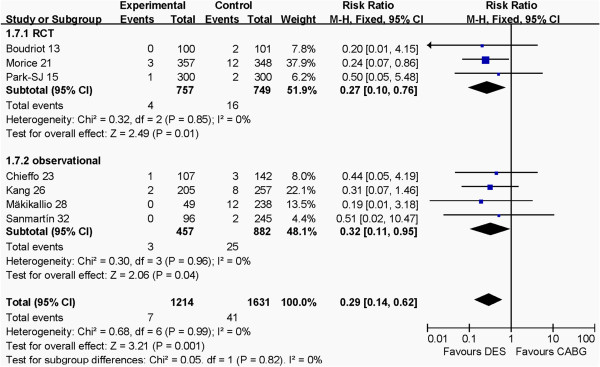
Comparison of DES and CABG for the outcome of cerebrovascular events at 1 year post-operation between RCT and observational groups.

**Figure 8:**
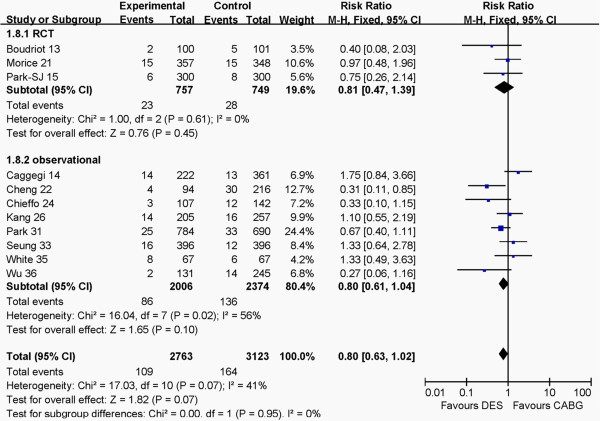
Comparison of DES and CABG for the outcome of death at 1 year post-operation between RCT and observational groups.

**Figure 9:**
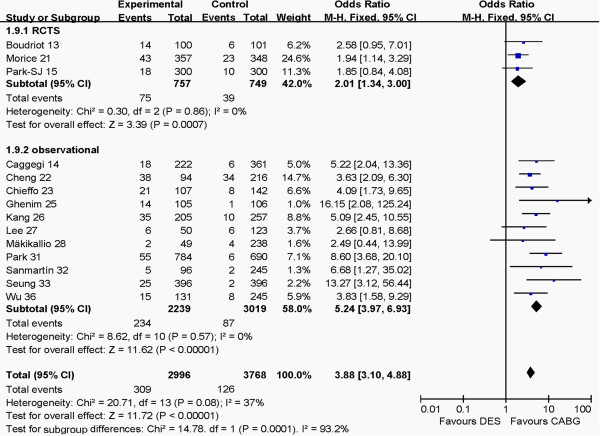
Comparison of DES and CABG for the outcome of revascularization at 1 year post-operation between RCT and observational groups.

The outcomes of RCT and observational groups at 1 year are shown in Figures 6, 7, 8, 9. Pooled effects showed that there were no different outcomes between RCT and observational groups in death, myocardial infarction, cerebrovascular events or revascularization. There were also no differences in both death and myocardial infarction for CABG and PCI in both RCT and observational groups (*P* >0.05 for each). The PCI group had higher revascularization risk than the CABG group (*P* <0.00001), whereas the CABG group had higher cerebrovascular events risk than the PCI group (*P* = 0.001) in the two groups.

### Sensitivity analysis

Sensitivity analyses were performed to assess the contribution of each study to the pooled estimate and by excluding individual studies one at a time and recalculating the pooled RR estimates for the remaining studies. Eliminating the studies with more than 300 patients or fewer than 100 patients in each group did not substantially change the pooled point estimate. Moreover, analysis of four RCTs separately did not also substantively alter the overall result of our analysis. Last but not least, performing transition of model also did not substantially change the pooled point estimate.

## Discussion

The results of the present meta-analysis showed that the early subtotal outcomes of death, cerebrovascular events and composite endpoint; death at 2, 4 and 5 years post-operation and composite endpoint at 1, 4 and 5 years post-operation, combined with their total outcomes, were lower risk in PCI than in CABG groups. There was no difference in the risk for the early outcomes of myocardial infarction, death at 3 years and composite endpoint at 2 and 3 years. Nevertheless, there was a lower risk for revascularization associated with CABG. There was no significant difference in death, myocardial infarction, cerebrovascular events or revascularization between RCT and observational groups.

Recently, three meta-analyses [10,12,37], including RCTs and observational studies, showed no significant differences in the safety between CABG and DES, and superiority of CABG to DES for repeated revascularization in patients with ULMCAD. A meta-analysis including 3,773 patients and follow-up of 3 years believed that PCI was emerging as an acceptable option. However, the PCI group in the meta-analysis was mixed with bare-metal stents and DES but did not compare DES with CABG separately, which might have led to the less robust results [37]. The meta-analysis by Lee et al. [10] included 8 clinical studies and 1 year follow-up. However, the number of patients in the CABG and DES groups was wrong in one study [38] and the total number of studies and patients was small, which may also have led to weak results. The meta-analysis by Zheng et al. [12] published in 2011 was heavily based on observational studies (13 observational studies and 2 RCTs) and a 5-year follow-up in the two groups, however, it abstracted and combined unadjusted risk estimates not only from randomized trials but also from observational studies, which did not strengthen the conclusion.

Two recent meta-analyses including a single RCT have been published. In one meta-analysis including three RCTs, Kajimoto et al. [9] showed that there was no significant difference in the risk of death and myocardial infarction in two groups but was superior to target vessel revascularization and major adverse cardiac and cerebrovascular events in CABG than in PCI group at 1 year. Therefore, they believed that CABG remains the standard of care for the treatment of left main coronary artery disease. However, the meta-analysis included a large power article [39] with 1,800 patients mixed with left main coronary artery disease and three-vessel coronary disease but not comparing the results of left main coronary artery disease in the two groups separately, which also affected the results.

The meta-analysis by Desch et al. [40] including four RCTs showed that there were no significant differences in the clinical endpoints of death and myocardial infarction between the PCI and CABG groups. While stroke was more frequent in surgical patients, the risk of repeated revascularization was higher in the PCI up to 2 years. Therefore they believe PCI to be useful only as an alternative to CABG in anatomically suited patients and with an increased risk of adverse surgical outcomes. However, the meta-analysis included an article [41] assessing mixed bare-metal stents and DES but not comparing DES with CABG separately, and the size of the study population was small.

In the present study, however, we exclude the articles that mixed left main coronary artery disease and three-vessel coronary disease but did not compare left main coronary artery disease in the two groups separately, or articles assessing mixed bare-metal stents and DES but not comparing DES with CABG separately, and we included more studies (four RCTs and 17 observational studies) and larger number of patients (total 8,413). Further, we performed the systematic review using a different method, which may be the reason for the different outcomes with the previous meta-analyses. We also performed the analysis of RCT and observational groups separately, there was no significant difference in death, myocardial infarction, cerebrovascular events or revascularization between RCT and observational groups. These also made our conclusion more robust.

### Quality of the evidence

Some of the evidence GRADE level was low because most of the included studies were poor quality. Seventeen studies were observational studies and were not performed with the method of randomization and allocation concealment, which might lead to selection bias and an exaggerated RR. Combined with not performing methods of blinding could result in performance, attrition and detection bias. These method limitations caused down grade of the quality of evidence. On the other hand, some differences in baseline characteristics among treatment groups might have an unknown influence on the estimated effects that would increase inconsistent results, and some trials in these groups had inconsistent results and high heterogeneity; all this also caused downgrade of the quality of evidence. Furthermore, only the articles in English were included in this analysis and we were unable to search for grey articles, which might be a source of potential publication bias in this study. The low quality of GRADE did not allow a robust conclusion for some groups in this population.

**Figure 10:**
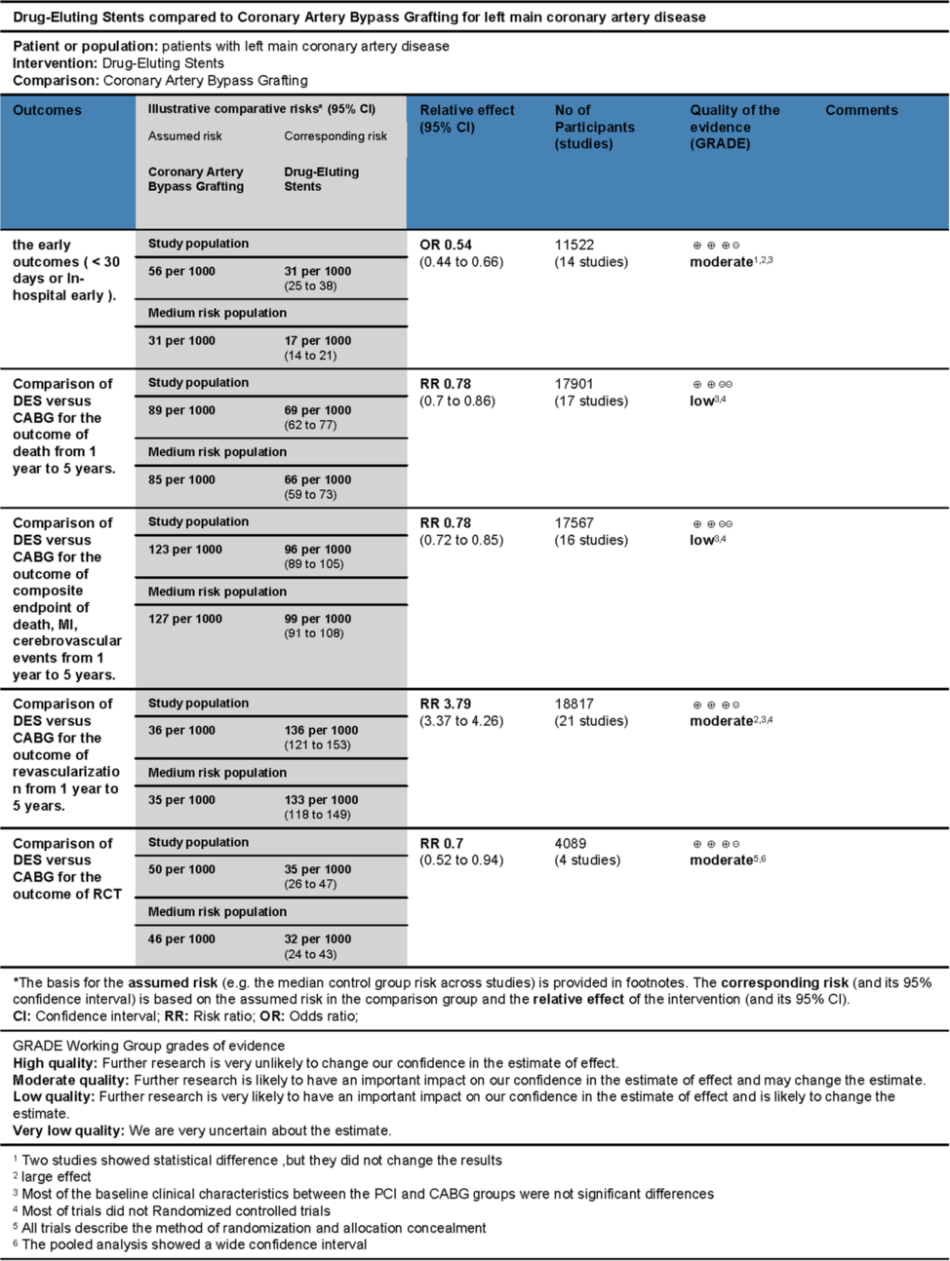
Summary of finding for the main comparison.

However, some total or subtotal RRs had a large effect. All RCTs describe the method of randomization and allocation concealment. These subgroups of RCT had consistent results and low heterogeneity, but the size of the study population of RCT was a bit small and the pooled analysis showed a wide CI. Therefore, some of the evidence GRADE level was moderate (Figure 10).

Other limitations should also be discussed in our study. Firstly, only four RCT were in the included studies in our meta-analysis, and two RCTs had duplicate patients and most types of data of outcomes in the two studies were repeated. Therefore, in the future, more randomized studies to compare DES with CABG in patients with left main coronary artery disease are necessary. What is more, many studies’ period of follow-up was short and only three observational studies [23,30,31] reported long-term follow-up (5 years). Therefore, more long-term results are necessary in the future.

## Conclusions

Our meta-analysis indicates that DES has a lower safety risk than CABG but is inferior to CABG for repeated revascularization in patients with ULMCAD in the 5 years after intervention. There was no difference in death, myocardial infarction, cerebrovascular events or revascularization between RCT and observational groups.

## Abbreviations

CABG: Coronary artery bypass grafting; CI: Confidence interval; DES: Drug-eluting stents; PCI: Percutaneous coronary intervention; RR: Risk ratio; ULMCAD: Unprotected left main coronary artery disease.

## Competing interests

The authors declare that they have no competing interests.

## Authors’ contributions

QL conceived the study, participated in the design, collected the data, performed statistical analyses, and drafted the manuscript. ZZ helped to collect the data and perform statistical analyses. RXY conceived the study, participated in the design, and helped to draft the manuscript. All authors read and approved the final manuscript.
